# Mechanisms and Control Strategies of Antibiotic Resistance in Pathological Biofilms

**DOI:** 10.4014/jmb.2010.10021

**Published:** 2020-12-07

**Authors:** Ying Luo, Qianqian Yang, Dan Zhang, Wei Yan

**Affiliations:** 1 Department of Pharmacy, Hangzhou Geriatric Hospital, Hangzhou 30022, P.R. China; 2 Department of Pharmacy, Affiliated Hangzhou First People's Hospital, Zhejiang University School of Medicine, Hangzhou 310006, P.R. China

**Keywords:** Biofilm, antibiotic resistance mechanisms, quorum sensing, control strategies

## Abstract

Bacterial biofilm is a community of bacteria that are embedded and structured in a self-secreted extracellular matrix. An important clinical-related characteristic of bacterial biofilms is that they are much more resistant to antimicrobial agents than the planktonic cells (up to 1,000 times), which is one of the main causes of antibiotic resistance in clinics. Therefore, infections caused by biofilms are notoriously difficult to eradicate, such as lung infection caused by *Pseudomonas aeruginosa* in cystic fibrosis patients. Understanding the resistance mechanisms of biofilms will provide direct insights into how we overcome such resistance. In this review, we summarize the characteristics of biofilms and chronic infections associated with bacterial biofilms. We examine the current understanding and research progress on the major mechanisms of antibiotic resistance in biofilms, including quorum sensing. We also discuss the potential strategies that may overcome biofilm-related antibiotic resistance, focusing on targeting biofilm EPSs, blocking quorum sensing signaling, and using recombinant phages.

## Introduction

Biofilms are protected microbial structures enclosed by a self-secreted extracellular matrix [1]. Bacteria biofilm is one of the most successful forms of life, being widely distributed in a diversity of environments [2]. All higher organisms (including humans) are colonized by microorganisms that form biofilms, mostly bacteria [3]. The ability of biofilms to escape the host immune system and resist antibiotics poses great health threats to patients [4, 5].

Biofilm formation contributes to drug resistance and inflammation, causing persistent infections in patients [6]. Biofilms also function as cell reservoirs that can repopulate infection sites after the release of suppression. For example, biofilm formation is a major concern for cystic fibrosis pneumonia caused by *Pseudomonas aeruginosa* and urinary tract infections caused by *Escherichia coli*, both of which are refractory to multiple antibiotics [7]. Therefore, basic research on biofilm formation and the mechanism of their resistance is of great clinical relevance. The objective of this review is to summarize the current research progress in antibiotic resistance mechanisms associated with bacterial biofilms and anti-biofilm strategies, which may greatly benefit patients who suffer from biofilm-related infections.

## Bacterial Biofilms

### Structure and Characteristics of Bacterial Biofilm

**Fig. 1:**
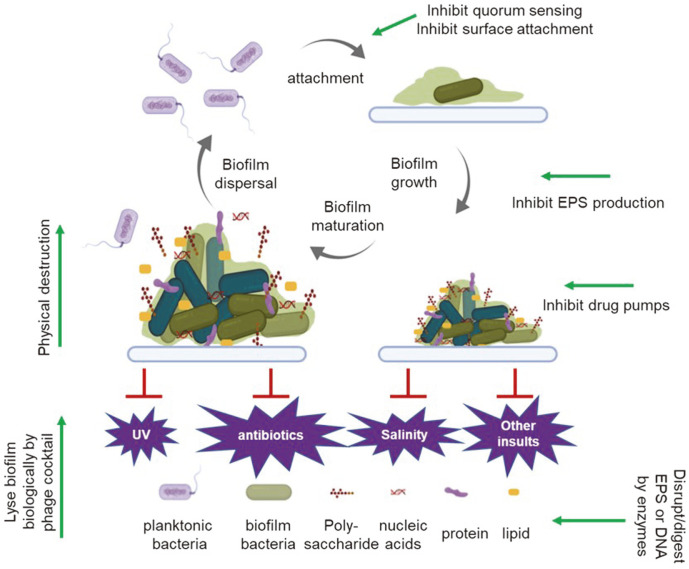
Structure and life cycle of bacterial biofilms. (1) Initial surface attachment of planktonic bacteria cells; (2) cell to cell adhesion and biofilm growth; (3) biofilm maturation; and (4) cell detachment and biofilm dispersal. Polysaccharides, nucleic acids, protein and lipids are major components of biofilms. The biofilm form of bacteria is resistant to many environmental insults, including UV light, antibiotics, and salinity. Anti-biofilm methods mentioned in this review are shown, indicated with green arrows at the locations where they act.

The physical scaffold of a biofilm is the matrix of extracellular polymeric substances (EPSs), self-secreted substances that keep bacterial cells in a contained structure and attach them to surfaces [8]. Most of the biomass of the biofilm is hydrated EPS rather than bacterial cells, which only make up between 2% to 15% of the total biofilm mass [9]. EPS is mostly composed of polysaccharides, proteins, lipids and extracellular DNA (eDNA) (Fig. 1) [9]. Enhanced antimicrobial resistance, nutrient capture and social cooperation are three main characteristic features of biofilms, and the EPS matrix underlies these important properties [2]. The structures of biofilms somewhat resembles tissues of higher organisms, which are structurally complex and highly heterogenous in gene expression [10], both contributing to the resistance mechanisms of biofilm.

### Life Cycle of Bacterial Biofilms

The transition from planktonic growth to biofilm is a complex and highly regulated process that follows a few steps (Fig. 1). The first step is the initial attachment of planktic bacterial cells to a surface, which often happens when cells are under environmental stress. The propelling structures, such as appendages, fimbriae and sex pili, have important roles in the initiation of biofilm [11]. The aggregation interactions can be either non-specific or ligand-receptor mediated [12, 13]. At this stage, single seeding cells are barely covered by EPS. Next, the seeded bacteria cells multiply and secret quorum sensing molecules that dictate the gene expression of the biofilm [14], which will be discussed in detail below. One of the most important changes is the increased secretion of EPS, which fills the spaces between bacterial cells and provide the structural rigidity. Rapid proliferation and aggregation of bacteria follow and increase the volume of the biofilm dramatically, until it adopts the signature morphology of a mushroom-like architecture [15]. The volume of a mature biofilm is made up mostly by EPS, as well as polysaccharides, lipids, protein and external DNA [16]. The matured structure is essential for the antibiotic resistance of enclosed bacteria. However, EPS not only blocks the access of environmental stresses but also limits the exchange of essential substances, causing the accumulation of metabolic products that may be toxic. At this stage, bacteria within the biofilm start the self-dissolving program by secreting EPS-digesting hydrolases. When EPS is digested, the embedded bacteria is released as free planktic cells, ready to seed new surfaces (Fig. 1). The planktonic cell-biofilm cycle is a major source of recurrent infections and chronic infections [6].

### Biofilm-Associated Infections

Hospital-acquired, nosocomial, and medical device-originated infections are the most common forms of biofilm-related infections [6]. Biofilm-associated chronic infections are often highly resistant to antibiotic treatment, and they are very common in nosocomial infections such as hospital-acquired infections with extremely high mortality rates [6]. Fungal and bacterial biofilms account for approximately 65%-80% of all hospital-acquired infections, in which the urinary tract, respiratory tract and bloodstream are the major locations of biofilm-associated nosocomial infections, because those are common locations for plantation of internal medical devices [17, 18]. Hence, biofilms pose a public health problem for patients needing indwelling medical devices, especially for those who are already immune-compromised [4]. Biofilms on medical devices are notoriously difficult to remove once they form. On one hand, biofilm formation is surprisingly quick and seemingly irreversible after initiation [19]. On the other hand, detached planktonic bacterial cells frequently recolonize and cause recurrent infections. With the increasing number of implantation surgeries, a better understanding of resistance mechanisms and counteracting strategies of biofilms-associated infections is urgently needed (discussed below).

## Mechanisms of Antibiotic Resistance in Biofilms

### Complex Structures of Biofilm a Natural Defense

EPS is not only critical for the architecture of biofilms, it is also a physical barrier that shields and protects embedded bacterial cells from environmental insults such as antibiotics and ultraviolet (UV) light [20, 21](Fig. 1). For example, the negatively charged polysaccharides can effectively block the penetration of the positively charged, aminoglycoside-class of antibiotics through binding [22]. The EPS barrier also delays the diffusion of small molecules such as hydrogen peroxide to bacterial cells within the biofilm. It has been shown that planktonic *P. aeruginosa* cells are very sensitive to hydrogen peroxide, while its biofilm form can survive in much higher concentration [23]. In addition, the EPS barrier decreases the rate of drug penetration, which buys crucial time for a second resistance mechanism, such as activation of beta-lactamase expression, to kick in [24].

### Heterogeneity of Biofilms Contributes to Resistance

The heterogeneity of the conditions inside the biofilm prevents the total eradication of all bacterial cells by certain insults. In a sense, biofilms ‘never put all their eggs in one basket’. For example, there are nutrient and oxygen gradients from top to the bottom of biofilms. With the decease of nutrients and oxygen, cells at the bottom have decreased metabolic activity and growth rate [25]. Counterintuitively, it is the more dormant cells who contribute most to antibiotic resistance. In fact, almost all antibiotics kill the fast-growing cells more effectively [26]. In addition, the protein expression profile of biofilm is very different and more diverse than planktonic cells, which may drive the bacteria resistance. For instance, *Pantoea agglomerans YS19*, a rice endophytic bacterium that forms multicellular biofilm-like structures [27], expresses high levels of the acid-resistant protein SPM43.1 in its biofilm form to combat harsh environmental conditions [28].

### Antibiotic Resistance through Quorum Sensing

Quorum sensing (QS) in bacteria is a mechanism of inter-cell communication using signal molecules, which are activated and secreted to coordinate community behaviors to defend against unfavorable environmental conditions. QS is a major mechanism for activation and formation of bacterial biofilms, negating the effectiveness of antibiotics against pathological bacteria [29]. Environmental factors such as antibiotics, pH, salt concentration, and nutrition deprivation modulate QS-mediated functioning in biofilms [30]. The feed forward mechanism amplifies QS signaling in the biofilm community [31]. When the concentration of the QS signal molecules reaches a threshold, synchronized behavior of bacteria is triggered, leading to social activities such as biofilm formation [32]. The QS signal is subsequently translated into cells to modulate the expression level of genes. These genes are important for cells to adjust to their environment, including genes that are involved in biofilm formation [33]. The secretion systems of bacteria are also highly regulated by QS signal, including the multidrug efflux pumps [34, 35] and EPS secretion systems [15, 36].

There are three major types of bacterial QS signal molecules, namely acyl-homoserine lactones (AHLs), oligopeptides, and autoinducers [37]. Other molecules such as indole have been reported to function as a QS molecules in several microorganisms [38, 39]. Gram-negative bacteria frequently utilize the diffusible AHLs as QS molecules, while gram-positive bacteria use peptide-based QS mechanisms [40]. A better understanding of QS signal molecules will provide critical information for the development of chemicals or methods for controlling biofilm. This has become a hot research area in the search for an effective molecule that can either neutralize QS molecules or compete with their receptors [41-43].

### Increased Efflux Pumps in Biofilm Resistance

An increasing number of studies at the molecular level have shown that enhanced efflux pumps are a common and critical mechanism of antibiotic resistance in biofilms [44]. This has been widely studied in the common pathogen *P. aeruginosa*. For example, Zhang and Mah discovered and characterized the PA1874-1877 cluster of genes, which are involved in the biofilm-specific antibiotic resistance [45]. PA1874-1877 are components of an ATP-binding cassette (ABC) transporter complex and are overexpressed in biofilms compared to planktonic cells and also contribute to biofilm-specific resistance [45].

Additionally, multidrug pumps such as MexAB-OprM and the MexCD-OprJ contribute to the antibiotic resistance of biofilms, and inhibition of those pumps restores the antibiotic susceptibility of multidrug-resistant *P. aeruginosa* strains [46]. Liao *et al*. showed that the MerR-like regulator BrlR plays an important role in antibiotic tolerance in biofilms, specifically through the upregulation of the MexAB-oprM and MexEF-oprN efflux pumps [47]. Moreover, the MexAB-OprM pump also contributes to ofloxacin resistance in *P. aeruginosa* biofilm [48]. Efflux pumps can also modulate quorum sensing responses, although the definitive relationship between the two remains unclear [49, 50]. Taken together, multidrug efflux pumps are critical players in antibiotic resistance in clinical biofilms.

Taken together, antibiotic resistance of biofilm is multifactorial, and a wide range of molecular mechanisms contribute to biofilm resistance. Each of the above-discussed mechanisms may only partially contributes to the increased antibiotic resistance. However, when they act in concert, those mechanisms ensure the survival of a biofilm community when facing aggressive antibiotic treatment regimens. While the resistance mechanisms of biofilms are essential for the survival of microbes, they present threats to the public health. It is critical to better understand those resistant mechanisms at molecular levels, which will make targeted therapies capable of overcoming the antibiotic resistance of biofilms.

## Strategies to Overcome Antibiotic Resistant in Biofilms

Due to the above-mentioned resistance mechanisms, bacterial biofilms are difficult to eradicate even with prolonged treatments. Biofilms remain a major problem during the treatment of chronic infections. Many strategies are currently under investigation, aiming to effectively eradicate biofilm-related infections. In this section of the review, we will discuss some of the emerging strategies to overcome antibiotic resistance (also see Table 1).

### Combine Physical Destruction and Antibiotic Treatment

Using ultrasound-targeted microbubble destruction (UTMD) in combination with antibiotics has been shown to improve the efficacy of antibiotics. For example, the use of UTMD with vancomycin significantly improves the efficacy of vancomycin against *Staphylococcus epidermidis* biofilms [51]. Also, the combination of surgical debridement followed by antibiotic treatment can be effective to restrict and prevent biofilm formation in a wound. Harrison-Balestra *et al*. showed that it only takes about 10 hours for bacteria to form biofilm in a wound, and the colonized biofilm can persist for a long period of time [52]. Although surgical debridement of chronic wounds can remove biofilms, clinical studies show that biofilms will reform and mature completely within 3 days. However, the planktonic bacteria after debridement are sensitive to antibiotics [53]. Thus, surgical debridement to destruct biofilms and expose bacteria in their antibiotic sensitive, planktonic form, is an effective and practical strategy to overcome biofilm antibiotic resistance. Control of biofilm by blocking biofilm-surface interactions has been well reviewed by Vuotto and Donelli [54].

### Target the Structure of Biofilm

The integrity of biofilm structure ensures the protection of bacteria embedded within. EPS-targeting strategies have been well explored for quite some time [1, 55]. EPS-targeting can be achieved by inhibiting EPS production or secretion, blocking EPS adhesins to surface, or degrading EPS directly. The EPS disrupting agents include those that inhibit synthesis and secretion of EPS, such as cyclic-di-GMP (cdGMP) and cyclic-di-AMP (cdAMP) [56]. More recently, small-molecule screens have identified several small molecules that inhibit EPS production, including those that inhibit glucosyltransferase [57] and pilus formation [58]. EPS-degrading enzymes such as glucanohydrolases and dispersin B can enzymatically digest the EPS of pathogenic oral biofilms [59]. Glycoside hydrolases have been shown to degrade *Staphylococcus aureus* and *P. aeruginosa* biofilms in chronic wounds in mouse models [60]. In addition, substances naturally secreted by commensal bacteria can potentially prevent the biofilm formation of other species. Esp, a serine protease secreted by *S. epidermidis*, reduces *S. aureus* biofilm formation and, more strikingly, it also destroys the preexisting *S. aureus* biofilm in vitro. More importantly, the Esp-secreting *S. epidermidis* eliminates the nasal colonization of *S. aureus* in vivo [61]. In addition, DNA enzymes such as DNase I have been shown to effectively disrupt biofilms, which is true in vitro and in vivo [62-64], and also makes sense because eDNA is a main component of an EPS matrix (Fig. 1).

### Block Quorum Sensing Signaling

Since QS plays a critical role in signaling biofilm formation, anti-QS agents can theoretically abolish QS signaling and prevent biofilm formation. Therefore, anti-QS agents may overcome antibiotic resistance caused by biofilm formation [29, 65]. Based on the mechanism of QS signaling, this approach can be achieved by blocking QS signal molecule production [66], neutralizing signal molecules [41, 67], blocking the receptors or inhibiting the signaling pathway [42, 43]. This QS-deactivating phenomenon is commonly referred to as quorum quenching (QQ). QQ methods include inhibition of QS signal molecule synthesis, sequestration of signal molecules, using receptor antagonist, and inhibition of targets in the QS signal transduction pathway [73]. The sequestration of signal molecules can be achieved by using chemicals, antibodies, or specific enzymes [74].

Substantial efforts have been made to develop anti-QS inhibitors, and a lot of new anti-QS inhibitors have been reported [29, 68]. Peptide-based quorum sensing modulators are actively being developed for controlling bacterial biofilm, and this approach appears to be more effective for gram-positive bacteria [69-71]. Two types of anti-QS chemicals are particularly promising, namely phytochemicals and plant by-products [72]. Researchers are also actively seeking natural compounds that possess anti-quorum sensing properties [43,72-74]. Anti-QS agents are mechanistically sound, promising a new class of agents to tackle biofilms.

### Biofilm Targeting Recombinant Phages

Bacteriophages, the bacterial viruses, are natural enemies of bacteria. Many bacteriophages also secrete depolymerases that degrade the EPS of biofilms, which makes them ideal for targeting biofilms [75]. To maximize the effect of phage against biofilms, multiple bacteriophages can be combined to formulate super phage mix or so-called ‘phage cocktail’ [76]. In addition, some strains of bacteriophages naturally contain a cocktail of depolymerases. For example, bacteriophage K, a polyvalent *Staphylococcus* phage, can lyse ten different *S. epidermidis* strains [77] and nine different *Staphylococcus* strains [78]. The super phage also digests vancomycin-resistant *S. aureus* strains and a few other methicillin-resistant *Staphylococcus* strains [78]. Taken together, bacteriophages are very promising tools that can be harnessed to control or even eradicate bacterial biofilm [79, 80].

### Other Strategies

In addition to the strategies discussed above, other ways to prevent or inhibit biofilms have been studied. For example, techniques using physics principals have been explored. Nanoparticles are currently being widely investigated as a novel treatment of infections caused by multidrug-resistant bacteria, and the anti-biofilm properties of nanoparticles are likely to be one of the mechanisms [81]. TiO_2_ (Titanium dioxide) nanoparticles have been shown to prevent biofilms formed by *S. aureus*, *S. epidermidis*, *P. aeruginosa*, *E. coli*, and *Candida abicans* [82]. Silver nanoparticles prevent the bacterial colonization of internal surfaces of medical devices [83]. The other physics-oriented approach the use of low intensity electrical current. Earlier studies have shown that weak continuous direct electric currents significantly increase the efficacy of the antibiotic in biofilm, a phenomenon known as ‘bioelectric effect’. Caubet *et al*. demonstrated that low intensity electrical current reduces the number of bacterial cells in the biofilm and hampers the growth of *E. coli* biofilm [84]. Niepa *et al*. demonstrated that cathodic electrochemical currents effectively kill persister cells in *P. aeruginosa* biofilm [85]. More recently, Dusane *et al*. showed that direct current could damage *P. aeruginosa* cells and disrupt the architecture of the biofilm [86]. In addition, antibodies that specifically target components of biofilm have been able to disrupt *Salmonella* Typhimurium biofilm [87]. Another way to combat biofilm is attenuating the production or effectiveness of biofilm virulence factors. The virulence factors include those that are responsible for the overall growth and that contribute to biofilm formation [88]. The small-molecule inhibition approach is currently being widely investigated to target bacterial biofilms [89]. Antibiofilm strategies in *Candida* [90] and *Escherichia* [91] were recently reviewed by Cavalheiro *et al*. and Verderosa *et al*., respectively.

In summary, many anti-biofilm strategies are currently being investigated, including targeting EPS, blocking quorum sensing, engineered bacteriophage, and combining antibiotics with other methods such as physical destruction, nanoparticles and electric currents (Table 1). Given biofilms are notoriously resistant to many antimicrobial monotherapies, the combination of antibiotics and alternative therapy has great potential to enhance the effectiveness of antibiotics in bacterial biofilm infections. The anti-biofilm strategies that we discussed above can be combined with traditional antibiotics treatment regimens to achieve better efficacy.

## Summary

In this review, we summarized the characteristics and life cycles of bacterial biofilms and clinically associated biofilms. We reviewed the current progress in biofilm research, focusing on the mechanisms by which biofilms become resistant to antibiotic treatment. We also reviewed the current basic and translational research studies on how to overcome antibiotics resistance associated with biofilm. Although there has been tremendous progress in both antibiotic-resistant mechanisms and corresponding strategies to override resistance, biofilm-associated infections remain a huge challenge. Further studies in both basic science and clinics are needed to eradicate biofilm-associated, antibiotic-resistant bacterial infections. Novel small molecules possess great potential to deliver concrete anti-biofilm treatment, especially those that can efficiently inhibit the formation of biofilm up front. High-throughput chemical screens should be employed to identify novel chemicals with such properties. Screening for chemicals that inhibit quorum sensing, EPS production and multidrug efflux pumps are sound and promising directions that should all be given more research attention in the future.

## Figures and Tables

**Table 1:** Methods to overcome antibiotic resistance in biofilms.

Strategy	Mechanism	Methods or agents	Target Microorganisms
Physical destruction	Physical destruction of biofilm structure	UTMD [51] Surgical debridement plus antibiotic [52, 53]	*S. epidermidis* *P. aeruginos*
Target the EPS	Inhibit EPS production or secretion	cdGMP and cdAMP [56] Small molecules such as glucosyltransferase inhibitor and pilicides [57, 58]	*S. aureus* *P. aeruginosa* *S. epidermidis* *Streptococcus*
	Degrade EPS	Glucanohydrolases and dispersin B [59, 60] Esp serine protease [61] DNase I [62-64]	*S. aureus* *P. aeruginosa* *S. epidermidis* *Streptococcus*
Block quorum sensing	Inhibit quorum sensing to prevent biofilm formation	Blocking QS signal molecule production [66] Neutralizing signal molecules by chemicals, antibodies, or specific enzymes [41, 67] Blocking the receptors or inhibit the signaling pathway [42, 43]	*P. aeruginosa* *B. Bosea* sp. *B. brevis* *A. caviae* Sch3
Recombinant phages	Use phage to lyse bacteria	Recombinant ‘phage cocktail’ [76] Nature strains of polyvalent bacteriophages [77, 78]	*Staphylococcus* strains *S. epidermidis* *S. aureus* *K. pneumoniae*
Others	Use chemical or physical principles	Nanoparticles: TiO2 [82] ; Silver [83]	*S. aureus*, *S. epidermidis*, *P. aeruginosa*, *E. coli*, and *C. abicans*;
		Electric currents [84-88]	*E. coli*, *P. aeruginosa*, *S. Typhimurium.*

Promising methods to overcome biofilm antibiotic resistance, with the underlying mechanism of action also shown.
